# Optimization of an ultrasound‐assisted extraction method to obtain gallic acid‐rich extracts from mango seed kernels

**DOI:** 10.1002/fsn3.4060

**Published:** 2024-04-12

**Authors:** Tuba Riaz, Zafar Hayat, Kinza Saleem, Kashif Akram, Hafeez Ur Rehman, Shafiq ur Rehman, Muhammad Azam

**Affiliations:** ^1^ IDRC Project Laboratory University of Veterinary and Animal Sciences Lahore Punjab Pakistan; ^2^ Department of Animal Sciences, College of Agriculture University of Sargodha Sargodha Punjab Pakistan; ^3^ Department of Food Sciences Cholistan University of Veterinary and Animal Sciences Bahawalpur Punjab Pakistan; ^4^ Department of Food Sciences University of Sargodha Sargodha Punjab Pakistan; ^5^ Institute of Microbiology and Molecular Genetics University of the Punjab Lahore Pakistan

**Keywords:** gallic acid, mango seed kernel, response surface methodology, ultrasound‐assisted extraction

## Abstract

Gallic acid is a widely recognized bioactive compound that falls under the category of secondary polyphenolic metabolites and is fairly found in mango fruit waste, specifically in mango seed kernel (MSK). This study aimed to adopt a green extraction approach to extract this valuable compound via ultrasound‐assisted extraction (UAE) without using organic solvents but only water to obtain hazard‐free extracts, and the cost of extraction can be minimal. pH (2–8), solvent ratio (20–60 mL/g), temperature (30–60°C) and time (30–60 min) of extraction were the independent variables used for extraction optimization. Single‐factor experiments to obtain working ranges for selected extraction variables were carried out. A central composite design using response surface methodology was used to determine the optimum condition to obtain the maximum yield of gallic acid from MSK. The optimized extraction conditions were 3.9 pH, 36.25 mL/g solvent ratio, and 39.4°C of extraction temperature for 21.3 min. As a result, the optimized yield was 5.76 ± 0.41 mg/g, which was comparably equal to and/or better than the other solvent extraction systems. The results showed that gallic acid could efficiently be extracted via UAE under these optimal conditions. It is safer than extraction systems involving hazardous solvents that can be feasibly used for its nutraceutical and therapeutic applications.

## Graphical abstract

This study aimed to adopt a green extraction approach to extract this valuable compound via ultrasound‐assisted extraction without using organic solvents but only water to obtain hazard‐free extracts, and the cost of extraction can be minimal. pH (2–8), solvent ratio (20–60 mL/g), temperature (30–60°C) and time (30–60 min) of extraction were the independent variables used for extraction optimization. A central composite design using response surface methodology was used to determine the optimum condition to obtain the maximum yield of gallic acid from mango seed kernel. As a result, the optimized yield was 5.76 ± 0.41 mg/g, comparably equal to and/or better than the other solvent extraction systems.

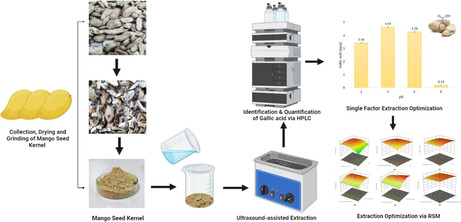

## 1 INTRODUCTION

Gallic acid is a commonly found polyphenol in almost all fruits, vegetables, and medicinal plants. The molecular mass of gallic acid is 170.12 g/mol. and is 1.1% water soluble at 20°C (Polewski et al., 2002). It has been extensively studied for its beneficial effects on human health, including antioxidant (Polewski et al., 2002), antibacterial and antiviral (Özçelik et al., 2011), anti‐inflammatory, antiallergic (Jung et al., 2010), anticarcinogenic, and antimutagenic, which are some of the major health and therapeutic benefits of gallic acid. It has been shown to scavenge free radicals and inhibit oxidative stress, which can help prevent cardiovascular disorders and many chronic diseases such as cancer and diabetes. In addition to its health benefits, gallic acid has been widely used in the pharmaceutical, cosmetic, and food industries as a natural preservative, food additive, and flavoring agent (Kahkeshani et al., 2019). Gallic acid is also used in the formulation of topical creams and lotions as it can combat skin oxidative stress due to its antioxidant and anti‐inflammatory properties (Monteiro e Silva et al., 2017).

Mango (*Mangifera indica*) by‐product waste (seed kernels and peels) contains polyphenols, including gallic acid, ellagic acid, mangiferin, and many others. Gallic acid is abundantly present in the form of gallates and gallotannins in all parts of mango but the highest in seeds (Gómez‐Caravaca et al., 2016). Mango seed kernel (MSK) is a resort of bioactive compounds and the major waste component, as it is estimated that 40%–50% of total mango production accounts for its agro‐waste (Castro‐Vargas et al., 2019), out of which more than 20% are only its seeds (Tesfaye, 2017). These seeds are usually thrown out in the environment, causing pollution and wastage of this precious agro‐waste that contains multiple phytochemicals. Notably, it contains 20%–21% tannins, 6–7 mg/g of gallic acid, 12–13 mg of coumarin, 7–8 mg of caffeic acid, 20–21 mg of vanillin, 4–5 mg of mangiferin, 10–11 mg of ferulic acid, 11–12 mg of cinnamic acid, and approximately 7–8 mg of unidentified compounds (Abdalla et al., 2007). MSK has nutraceutical potential and can be extracted from MSK using a safer, low‐cost, and less time‐consuming method that produces a final extract containing comparable amounts of gallic acid. Since gallic acid is found in mango, especially in MSKs, its extraction is an important aspect of its utilization. It can effectively be extracted using various modern extraction techniques like pulse electric field extraction, microwave‐assisted extraction, supercritical fluid extraction, ultrasound‐assisted extraction (UAE), etc. (Linares & Rojas, 2022). These methods have been used to extract different bioactive compounds from MSKs with minimal degradation or loss of bioactivity, but except UAE, other methods require specific space and installations for working, whereas the UAE is a benchtop‐friendly technique that requires minimal solvents for extraction and gives higher yields (El Maaiden et al., 2023). It can be a useful technique for the extraction of gallic acid from MSKs due to its effectiveness, simplicity, and high extraction yield. It is also an efficient, cost‐effective, and accessible approach that needs a simple setup and has the ability to work on multiple samples at once, ultimately lowering expenses and enhancing productivity (Aznar‐Ramos et al., 2022). Hence, UAE has been reported as an efficient extraction method for mango waste (Borrás‐Enríquez et al., 2021). It is an effective method to extract bioactive compounds from plants and agro‐waste (Zou et al., 2014). Ultrasound waves produce a mechanical effect that enables enhanced solvent penetration into tissues, and the solute diffuses quickly due to an increase in surface area between liquid and solid phases (Tiwari, 2015; Zou et al., 2013, 2014). That is why the UAE has been effectively utilized as an extraction method for phytochemicals or nutraceuticals from plants.

Many kinds of research have been carried out on the extraction of polyphenols from MSK by applying UAE. Castañeda‐Valbuena et al. (2021) optimized the extraction of phenolic compounds from mango by‐products through central composite design (CCD) via UAE and found a higher concentration of phenolic compounds as the dry matter basis per gallic acid equivalent (GAE) was 121.66 mg/g in MSK, extracted with less than 80% ethanol. Another study implemented the use of a CCD through the UAE to extract MSK in 49% ethanol to explore its potential as an antioxidant, and it was observed that the phenols extracted were mostly gallic acid and its derivatives (Ojeda et al., 2022). Similarly, a study found that 672 mg GAE/100 g of total polyphenols can be extracted from MSK by using 50% ethanol in 20 min through the UAE (Borrás‐Enríquez et al., 2021). Anta et al. (2020) investigated the effect of solvent mixtures (methanol:acetone:water) along with ultrasound amplitude on the yield of phenolic compounds from MSK via UAE and reported the extraction yield of phenolics as 71.35 mg GAE/g. Furthermore, a quaternary solvent system to extract phenolics from MSK via solid–liquid extraction was reported and concluded that ethanol, methanol, acetone, and water (3:3:3:1) can efficiently extract up to 78.24 mg/g of phenolics (Cabajar et al., 2021). The variation in the reported phenolic contents may be attributed to factors such as the type of plant, location of cultivation, extraction process, and the solvent utilized for extraction (Abdalla et al., 2007; Dorta et al., 2013; Soong & Barlow, 2006).

The utilization of higher levels of organic solvent for extraction is a concern as their utilization is hazardous. To rule this out, green extraction techniques have been adopted that refer to environment friendly and sustainable methods of extracting natural compounds that reduce or eliminate the use of hazardous solvents and minimize waste generation (Chemat et al., 2019). Several principles of green extraction have been developed, such as the use of viable plant resources and varieties, water as an alternative solvent, reduction in energy consumption, favoring safe, less time‐consuming processes and aiming for a biodegradable and non‐denatured extract from plants free of any contaminants (Chemat et al., 2012). These are also referred to as cold extraction techniques, where the stability of the extracted compounds is not compromised and the time or energy for extraction is minimized (Tiwari, 2015). However, the involvement of solvents in extraction such as methanol, ethanol, acetone, and their aqueous combinations has been a prominent feature of extracting bioactive compounds from plants (Boeing et al., 2014). A study used different solvents to extract polyphenols from MSK. Pure methanol and water had similar results, suggesting that water alone can extract most phenolic compounds (Ekorong Akouan Anta et al., 2018). Regardless of the extraction efficiency of organic solvents, water has remained a mostly used solvent in industries because it is non‐toxic, inexpensive, and environment friendly. With a combination of modern extraction methods, water can serve as the greenest solvent in order to obtain sustainable and safe nutraceuticals (Alonso‐Riaño et al., 2020).

Response surface methodology (RSM) is an innovative technique for optimization purposes, introduced by Box and Wilson (1951). RSM involves creating a model that relates yield to independent variables and using statistical analysis to optimize the variables for maximum yield. It evaluates multiple variables and requires fewer trial interactions (Liyana‐Pathirana & Shahidi, 2005). This approach not only provides a broader image of the effects of various factors but also aids in identifying the region where extraction optimization is done (Jang et al., 2017). In this line, a previous study was conducted by our research team to optimize the extraction of gallic acid rich extracts from MSK through RSM and obtained promising yields of gallic acid with 19.4% ethanol, which yielded comparable gallic acid to that of higher levels of solvent extraction systems (Hayat et al., 2023). So, this study was executed to evaluate the effect of the complete elimination of ethanol and explore the potential of water (the greenest solvent) to extract gallic acid from MSK, consequently exploring a possible cost‐effective way for the valorization of this waste.

## 2 MATERIALS AND METHODS

### 2.1 Sample material

During the peak production and processing season of mangoes, MSKs were obtained from a juice factory located in District Sargodha, Pakistan. To ensure the purity of the seeds, a thorough washing was conducted to eliminate any remaining residues of pulp. The seed coat was then manually cut using a sharp knife to extract kernels from the seed coat. The kernels were further cut into smaller parts and dried under shade until they reached a 10% moisture level. To maintain the quality of the samples, kernels were ground and stored in plastic sealed bags, which were kept at 4°C until ready to be used.

### 2.2 Extract preparation via UAE

Extracts were prepared according to the methodology of Hayat et al. (2023), with minor changes in finalized parameters including pH of the solvent (distilled water), solvent ratio, extraction temperature, and time. The extraction was executed in an ultrasonic bath (E30H, Elma, Germany) with a frequency of 37 kHz, equipped with an adjustable time and temperature controller. Water, being the “the greenest solvent,” was used after distillation at different pH levels. MSK powder (pre‐optimized mesh size 40 mm) was weighed precisely and mixed in the extraction solvent. The pH of the solvent was set by using hydrochloric acid and sodium hydroxide and monitored through a pH meter (Milwaukee). After proper mixing, the sample was put into an ultrasonic bath and exposed to pre‐determined parameters outlined in Section 2.3.1. Following the extraction through an ultrasonic bath, filtration was done with Whatmann filter paper. The resulting filtrates were then concentrated in a water bath (Memmert, Germany) at a temperature of 50°C, and HPLC analysis was performed to determine the gallic acid content of the extracts.

### 2.3 Experimental design

With the goal of studying factors interaction with each other, operating conditions via RSM were optimized by selecting the CCD method.

#### 2.3.1 Single‐factor experiments

Single‐factor experiments were performed to select the parameters of the extraction variable for RSM. The effect of pH of the solvent, solvent ratio, extraction time, and temperature was analyzed by keeping gallic acid yield as a response. The extracts were prepared using UAE, and the extraction was evaluated by setting the pH of the solvent at 2, 4, 6, and 8; solvent ratios at 20, 30, 40, 50, and 60 mL/g; temperature at 30, 40, 50, and 60°C; and extraction time at 30, 40, 50, and 60 min. The extractions variables were set at pH 4 and 50°C, 30 mL/g and 40 min for temperature, solvent ratio, and time, respectively, when not evaluated. HPLC analysis was performed to determine the gallic acid yield. Based on the results of single‐factor experiments, the parameters for the extraction variables were chosen.

#### 2.3.2 Multiple factor experiment

Four independent variables, pH (*X*_1_), solvent ratio (mL/g, *X*_2_), temperature (°C, *X*_3_), and time (min, *X*_4_), were utilized to optimize extraction parameters for gallic acid yield. Each independent variable has three levels—upper, middle, and lower—based on the results of single‐factor experiment. The basis of optimization was uncoded and coded levels. Three levels were selected based on a preliminary study against each independent variable and are presented in Table 1. This resulted in 27 experimental runs with three replications at center points to determine the method's repeatability index. To express the yield of gallic acid adequately, a polynomial regression model of second order was employed.

**TABLE 1:** Coded and uncoded levels of extraction parameters obtained by preliminary experiments.

Factors	−1	0	+1
pH	3	4	5
Solvent ratio (mL/g)	20	30	40
Extraction temperature (°C)	30	40	50
Extraction time (min)	30	40	50

### 2.4 HPLC analysis

After ultrasonic extraction, the extracts were filtered and concentrated, and to remove lipids and waxes, *n*‐hexane was used (Merck) three times, 5 mL each. A 0.45 μm syringe filter was used to filter the extracts for analysis (Ramirez et al., 2014). The phenolic profile was achieved by an Agilent HPLC equipped with a VWD detector. The analysis was performed on a Zorbax Eclipse plus C18 analytical 4.6 × 150 mm 5 μm (manufactured by Agilent, USA). The mobile phase consisted of a mixture of distilled water (DW) and formic acid (Merck) in a ratio of 99:1, v/v (solvent A), and a combination of acetonitrile and formic acid in a ratio of 99:1, v/v (solvent B). In a linear gradient, a flow rate of 0.6 mL/min following the scheme, *t* in minutes, %B: (0; 0%), (5; 20%), (10; 50%), (15; 100%) and (20; 0%) was used. Chromatograms were recorded at a temperature of 25°C with an injection volume of 20 μL using a wavelength of 280 nm. The quantification of gallic acid was based on the comparison of the peak area with a gallic acid standard curve (Sigma Aldrich). In the end, results were exhibited as the mean value of the assay for each experiment, which was performed in triplicate (Hayat et al., 2023).

### 2.5 Statistical analysis

SPSS (version 25) was used to compare means and significance levels (*p* < .05) of triplicate analysis of single‐ and multiple‐factor extraction through ANOVA. Design Expert Software, version 12, from Stat‐Ease, Inc., Minneapolis, MN, USA, was employed for data analysis in model building, predicting values, and illustrating the independent variable impact. Response variables were presented in a three‐dimensional graph. The best‐fitted model was determined by calculating a regression equation with a non‐significant lack of fit and pure error. The Fisher value test, lack of fit, and coefficient of determination *R* ^2^ were utilized to determine the quality and adequacy of the model.

## 3 RESULTS AND DISCUSSION

### 3.1 Single‐factor experiment results

#### 3.1.1 Effect of pH

**FIGURE 1:**
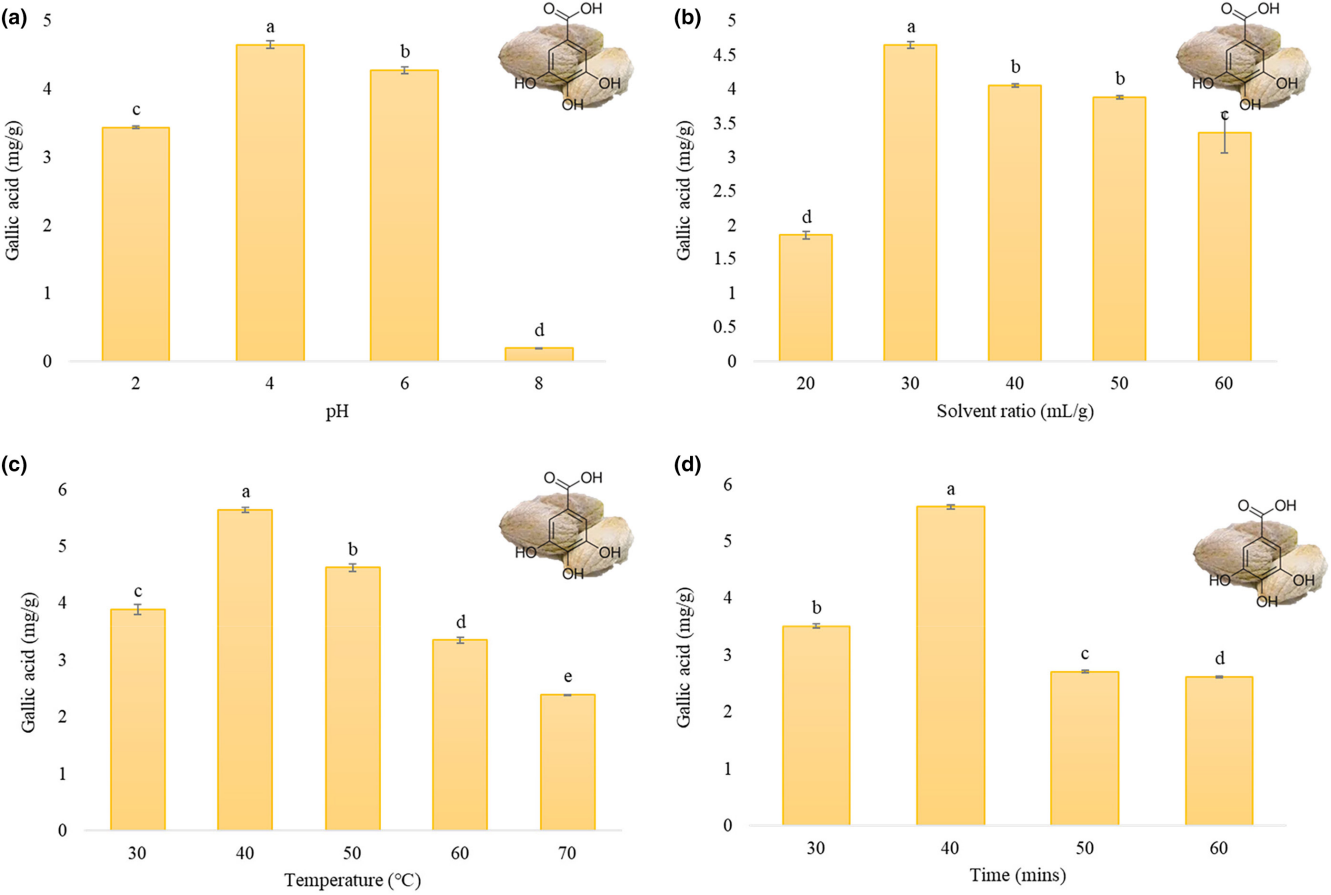
Extraction parameters effect on the yield of gallic acid from mango seed kernel: (a) pH; (b) solvent ratio (mL/g); (c) extraction temperature (°C); (d) extraction time (min).

The analysis showed that the gallic acid yield decreased with an increase in pH. At pH 4, the highest gallic acid yield was observed as 4.65 ± 0.05 mg/g, which gradually declined at pH 6 and 8, as given in Figure 1a. A similar effect of pH was reported in a study conducted on the green extraction of mango peels. The phenolic content increased up to pH 4 but started to decline as the pH rose higher than 4 (Tunchaiyaphum et al., 2013). The extraction of bioactive compounds is greatly influenced by the pH of the solvent, as the nature of the compounds has a direct link with the pH of the solvent. With the decrease in pH of the solvent, the solubility of the compounds, which are basic in nature, increases. With an increase in solubility, the concentration of extractable compounds also increases (Jenke, 2014). As the pH of gallic acid ranges between 7 and 10 (Eslami et al., 2010), it can be assumed that the pH of DW at four increased the solubility of gallic acid, which led to its enhanced extraction from the sample material. Therefore, the pH of DW at four was selected as the center point for extraction optimization.

#### 3.1.2 Effect of solvent ratio

The solvent ratios analyzed were 20, 30, 40, 50, and 60 mL/g, while other factors were constant as 4 pH, 40 min time, and 50°C extraction temperature. Figure 1b shows the yields of gallic acid observed. The rise in the liquid ratio increases the extraction yield to a certain level, as the ratio is the main driving force (Li et al., 2005). It was observed that the gallic acid yield was highest at 30 mL/g (4.64 ± 0.05 mg/g), which gradually decreased with the higher liquid ratios, as similar results were observed in a study conducted on polyphenol extraction from mango waste (Kaur et al., 2022). However, a few studies reported ratios higher than 30 mL/g as suitable for the extraction of bioactive compounds from mango by‐products (Castañeda‐Valbuena et al., 2021; Tunchaiyaphum et al., 2013). Similar extraction results were observed in optimization studies of gallic acid by using ethanol and UAE (Hayat et al., 2023), where solvent ratios higher than 30 mL/g tend to reduce gallic acid content. Hence, a small solvent ratio leads to a comparatively lower extraction yield. The rise in gallic acid yield in the present study is in line with the mass transfer principle, which is determined by the concentration gradient of the sample and solvent, especially when the solvent ratio is moderate to high (Tunchaiyaphum et al., 2013; Zou et al., 2013, 2014). Increasing the liquid flow rate and temperature can improve the extraction degree, as it is also important to balance to avoid leaving a solute or extraction solvent in the material.

#### 3.1.3 Effect of extraction temperature

Temperatures ranging from 30 to 70°C were evaluated for the extraction, and the results can be seen in Figure 1c. The graph depicts that there is a trend of increase in gallic acid yield when the temperature goes from 30 to 40°C, making it the highest among all the test levels of extraction temperature. In general, the ability of solvents to penetrate cells increases with higher extraction temperatures, leading to improved desorption and solubility of compounds from the cells and increased yield. Mostly, the bioactive compounds get denatured at high temperatures (Setyaningsih et al., 2016), as indicated by the results that the yield of gallic acid from MSK was decreased from 40 to 70°C. The maximum solubility and desorption equilibrium for gallic acid extraction were achieved at a temperature of 40°C, resulting in a yield of 5.64 ± 0.04 mg/g. 35 to 45°C was reported to be an efficient temperature range to extract phenolic compounds from mango waste (Cabajar et al., 2021; Kaur et al., 2022). Higher temperatures can improve the yield of plant extracts by increasing the permeability of cell walls and reducing solvent viscosity. However, excessively high temperatures can negatively impact the quantity and quality of extracted phenolic compounds (Zou et al., 2013). To balance both factors, an extraction temperature of 40°C was selected for multiple factor experiments.

#### 3.1.4 Effect of extraction time

Experiments were performed using different time durations (30, 40, 50, and 60 min). The results are shown in Figure 1d. A gradually higher yield was observed from 30 to 40 min and then started to decline as the ultrasonication continued to 50–60 min. At 40 min, the maximum yield was achieved, which was 5.61 ± 0.03 mg/g. The results are in agreement with the study reporting the optimum time of 41 min to gain maximum phenolic contents from MSK through the UAE (Anta et al., 2020). The findings confirm that ultrasonication accelerates the equilibrium coefficient between the solvent and cell walls of plants, resulting in a faster dissolution of target compounds during extraction. This is a significant advantage of the UAE over conventional extraction methods (Tabaraki & Nateghi, 2011). Exposure to ultrasonic waves and high temperatures for an extended period of time can increase the likelihood of the degradation of bioactive compounds in plant material (Gao et al., 2022). In these results, high extraction times reduced the yield. Therefore, the optimal time chosen for extraction was 40 min.

### 3.2 Gallic acid optimization via RSM

In the present study, gallic acid was optimized by using CCD via RSM. The gallic acid yields observed in all 27 extraction combinations are given in Table 2. The total number of runs generated was 27, so extracts were prepared according to the variables. The yield ranged from 0.43 ± 0.04 to 5.82 ± 0.03 mg/g. The highest gallic acid yield was observed at the extraction conditions of *X*_1_ = 3, *X*_2_ = 40 mL/g, *X*_3_ = 50°C and *X*_4_ = 30 min. Experimental data on gallic acid content were checked through multiple regression analysis by following Equation 1, where *Y* is the response and *X*_1_, *X*_2_, *X*_3_, and *X*_4_ are the factors for extraction optimization.

(1) $$\begin{array}{c} Y=5.28+0.2025X_{1}+0.6967X_{2}+0.1842X_{3}+0.2117X_{4}\\ \;-0.2988X_{1}X_{2}+0.3175X_{1}X_{3}+0.0225X_{1}X_{4}-0.0300X_{2}X_{3}\\ \;-0.4975X_{2}X_{4}+0.1137X_{3}X_{4}-0.3660X_{1}^{2}-0.9548X_{2}^{2}\\ \;-0.1973X_{3}^{2}-0.0885X_{4}^{2} \end{array}$$

**TABLE 2:** Experimental design using CCD on the gallic acid yield of mango seed kernel.

Run order	*X*_1_ pH	*X*_2_ Solvent ratio (mL/g)	*X*_3_ Extraction temperature (°C)	*X*_4_ Extraction time (mins)	*Y* ^a^ Gallic acid (mg/g)
1	3 (−1)	20 (−1)	50 (+1)	50 (+1)	2.82 ± 0.08
2	4 (0)	30 (0)	40 (0)	20 (−2)	4.99 ± 0.03
3	5 (+1)	40 (+1)	30 (−1)	50 (+1)	3.62 ± 0.05
4	4 (0)	30 (0)	60 (+2)	40 (0)	4.97 ± 0.04
5	4 (0)	30 (0)	40 (0)	40 (0)	5.29 ± 0.05
6	5 (+1)	40 (+1)	50 (+1)	30 (−1)	4.63 ± 0.05
7	4 (0)	50 (+2)	40 (0)	40 (0)	2.73 ± 0.05
8	3 (−1)	20 (−1)	30 (−1)	50 (+1)	2.25 ± 0.09
9	4 (0)	30 (0)	40 (0)	40 (0)	5.45 ± 0.06
10	6 (+2)	30 (0)	40 (0)	40 (0)	4.43 ± 0.05
11	3 (−1)	40 (+1)	30 (−1)	30 (−1)	4.71 ± 0.08
12	5 (+1)	40 (+1)	50 (+1)	50 (+1)	3.12 ± 0.04
13	4 (0)	10 (−2)	40 (0)	40 (0)	0.43 ± 0.04
14	5 (+1)	20 (−1)	30 (−1)	50 (+1)	3.19 ± 0.02
15	5 (+1)	20 (−1)	30 (−1)	30 (−1)	3.25 ± 0.11
16	3 (−1)	40 (+1)	50 (+1)	50 (+1)	4.56 ± 0.29
17	3 (−1)	40 (+1)	30 (−1)	50 (+1)	2.89 ± 0.05
18	5 (+1)	20 (−1)	50 (+1)	30 (−1)	3.07 ± 0.02
19	4 (0)	30 (0)	20 (−2)	40 (0)	4.25 ± 0.06
20	5 (+1)	40 (+1)	30 (−1)	30 (−1)	5.66 ± 0.10
21	2 (−2)	30 (0)	40 (0)	40 (0)	3.44 ± 0.07
22	4 (0)	30 (0)	40 (0)	40 (0)	5.11 ± 0.02
23	3 (−1)	20 (−1)	50 (+1)	30 (−1)	2.57 ± 0.09
24	5 (+1)	20 (−1)	50 (+1)	50 (+1)	3.85 ± 0.12
25	3 (−1)	20 (−1)	30 (−1)	30 (−1)	1.89 ± 0.04
26	4 (0)	30 (0)	40 (0)	60 (+2)	5.10 ± 0.05
27	3 (−1)	40 (+1)	50 (+1)	30 (−1)	5.82 ± 0.03

^a^ Gallic acid yield is expressed as the mean ± SD.

**FIGURE 2:**
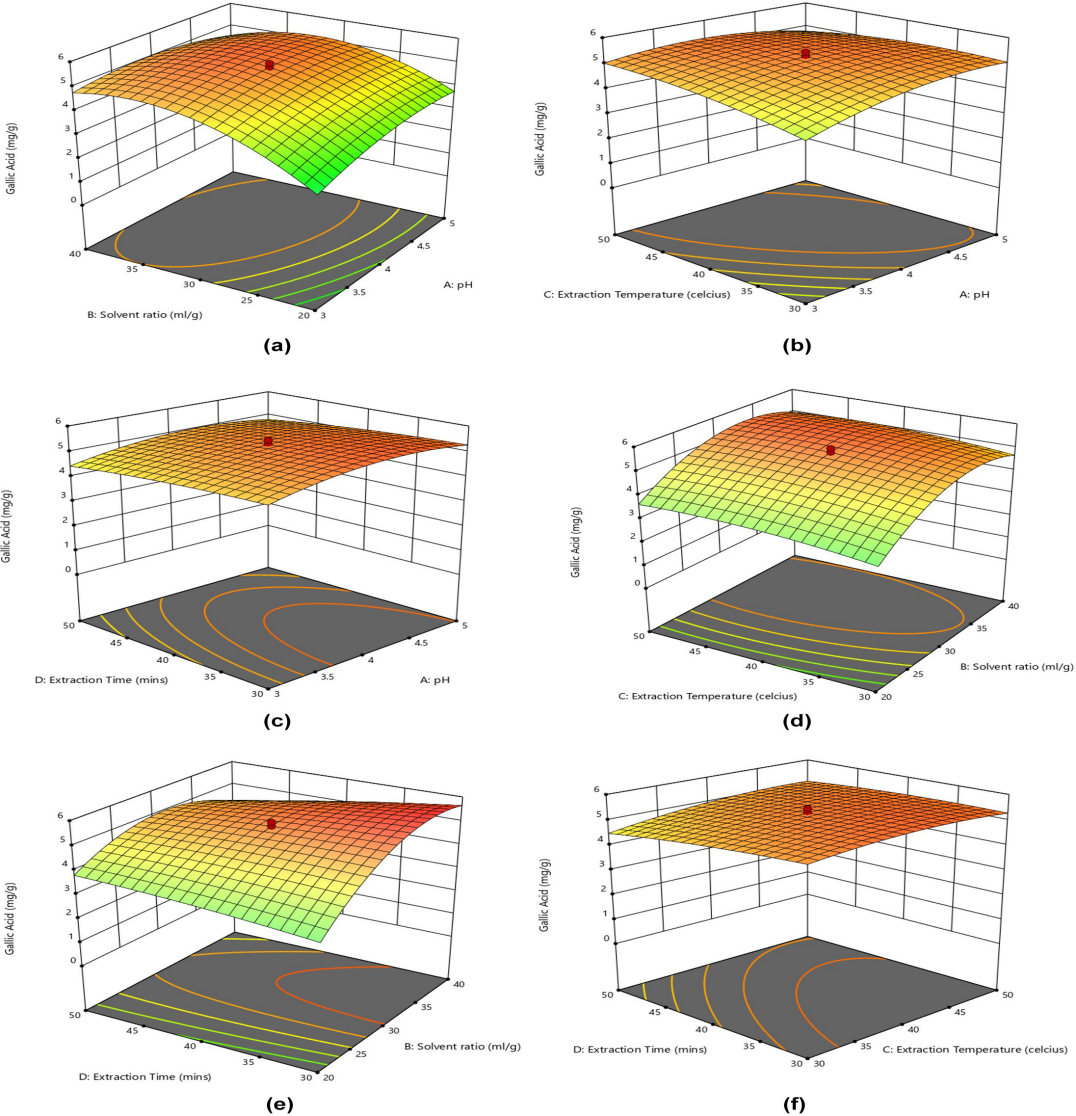
Response surface plots of gallic acid yield from MSK as effected by pH, solvent ratio, extraction temperature, and extraction time using UAE: (a) pH and solvent ratio; (b) pH and extraction temperature; (c) pH and extraction time; (d) extraction temperature and solvent ratio; (e) extraction time and solvent ratio; (f) extraction time and extraction temperature.

When subjected to UAE frequency and high temperature for an extended duration, plant cells undergo acoustic cavitation, leading to their rupture and the release of their contents into the surrounding water. However, at certain levels of pH with extraction temperature and time exposure, the gallic acid yield is enhanced; after that level, it starts to decrease due to the heat sensitivity of bioactive compounds. The three‐dimensional surface plots obtained by the model are shown in Figure 2.

The plots exhibited optimized values that showed the interaction between the variables explored in the optimization process. The appearance of the plot helps in identifying these interactions between variables, as the circular one exhibits an insignificant interaction, while an elliptical shape depicts a significant interaction between variables (Aware et al., 2019). Solvent ratio and extraction time showed a positive interaction toward gallic acid yield optimization, which means that the single‐factor experiment ranges were quite adequate. A notable enhancement in gallic acid yield was observed to a level of 36.25 mL/g solvent ratio, while an increase in the pH of the solvent depicted a slight reduction in the yield of gallic acid. These findings are in agreement with the studies reporting similar interactions (Jenke, 2014; Kaur et al., 2022; Li et al., 2005; Tunchaiyaphum et al., 2013), whereas the extraction time interaction with other factors was different. A significant interaction of solvent ratio with extraction time (*X*_2_*X_4_) was observed (Table 3), but it did not contribute to increasing gallic acid yield in the case of pH (*X*_1_*X_4_) and temperature (*X*_3_*X_4_).

**TABLE 3:** ANOVA for the fitted quadratic model.

Source	Sum of square	Mean square	*F*‐value	*p*‐value
Model	42.94	3.07	18.11	<.0001
pH	0.9841	0.9841	5.81	.0329
Solvent ratio	11.65	11.65	68.77	<.0001
Extraction temperature	0.8140	0.8140	4.81	.0488
Extraction time	1.08	1.08	6.35	.0269
pH * solvent ratio	1.43	1.43	8.43	.0132
pH * Extraction temperature	1.61	1.61	9.52	.0094
pH * Extraction time	0.0081	0.0081	0.0478	.8306
Solvent ratio * Extraction temperature	0.0144	0.0144	0.0850	.7756
Solvent ratio * Extraction time	3.96	3.96	23.38	.0004
Extraction temperature * Extraction time	0.2070	0.2070	1.22	.2906
pH^2^	2.86	2.86	16.87	.0015
Solvent ratio^2^	19.45	19.45	114.81	<.0001
Extraction temperature^2^	0.8304	0.8304	4.90	.0469
Extraction time^2^	0.1672	0.1672	0.9873	.3400
Lack‐of‐fit	1.97	0.1975	6.83	.1345

Table 3 presents the ANOVA findings for the regression equation. A lack of fit was assessed to confirm the model's suitability. A lack of significance (*p* > .05) indicated a good fit with the experiment. Accurate measurement of the signal‐to‐noise ratio is paramount, and a ratio greater than 4 is preferred. The current study showed a ratio of 17.744, which is a clear indication of a definite design space and confirms the precision of the model. The points plotted were tightly clustered around the diagonal line, indicating a robust correlation between experimental and predicted results and a superior model fit. The predicted *R* ^2^ value of .7442 is consistent with the adjusted *R* ^2^ value of .9021. A quadratic polynomial equation was established to quantify the relationship between the extraction parameters and the response. By solving the regression equation, optimal values of extraction variables were achieved. After running the experiment on design expert software, the common regions for optimal conditions of gallic acid extraction from MSK were obtained as 3.9 pH, 36.25 mL/g solvent ratio, extraction temperature at 39.4°C and 30 min of extraction with the corresponding *Y* = 5.78 ± 0.21 mg/g.

**FIGURE 3:**
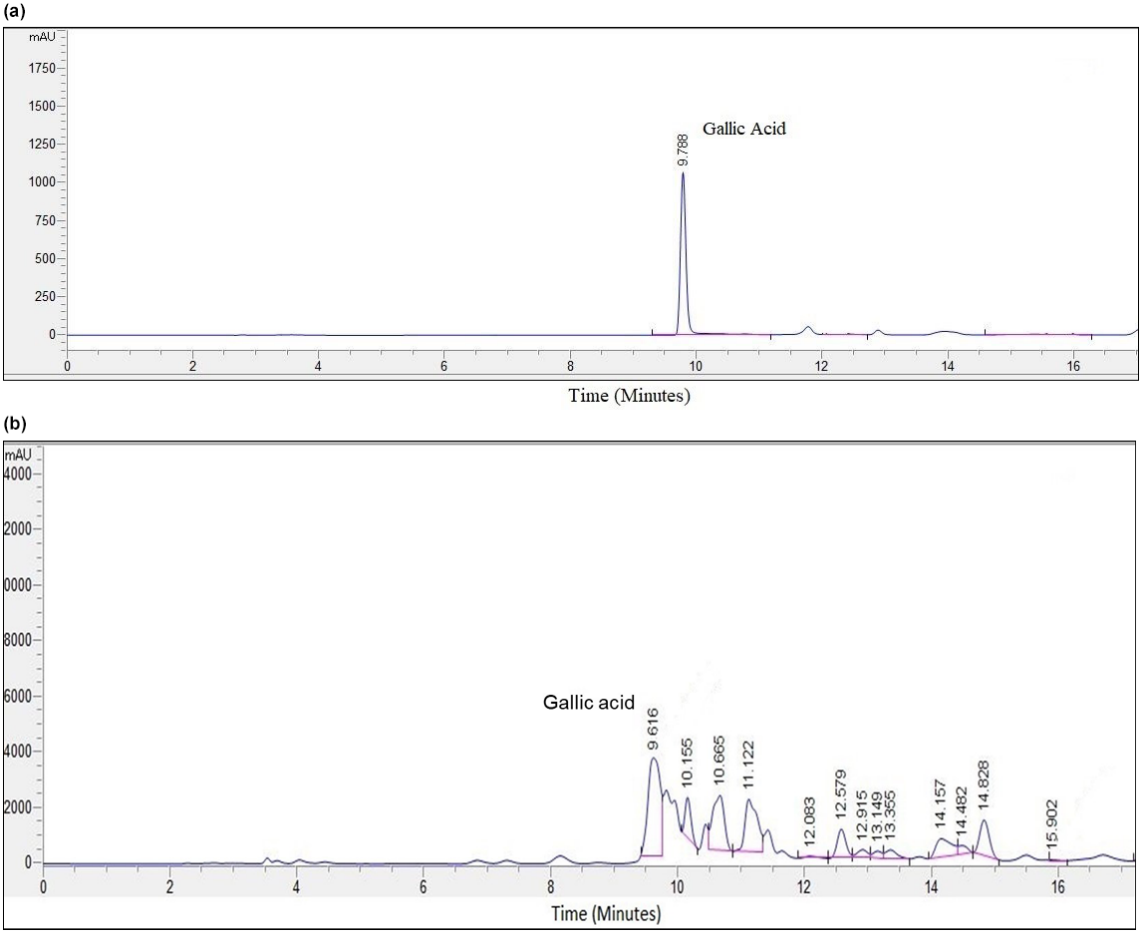
HPLC chromatogram of (a) gallic acid standard and (b) MSK extract obtained from optimized extraction conditions.

To confirm the optimized conditions, tests were conducted in triplicate using the optimized parameters. The chromatograms for the gallic acid standard and optimized MSK extract are presented in Figure 3a,b, respectively. The yield of gallic acid obtained was 5.76 ± 0.41 mg/g, indicating that the experimental data was accurately modeled and the extraction process was effectively optimized (Table 4). Along with gallic acid, some other bioactive compounds were also detected in the optimized extracts, as shown in Figure 3b. However, those compounds identified, such as chlorogenic acid and caffeic acid, were in trace amounts at 0.54 and 0.35 mg/g, respectively.

**TABLE 4:** Validation of optimum conditions with predicted and experimental values of gallic acid.

Optimum conditions				Extraction yield	
pH	Solid‐to‐liquid ratio (mL/g)	Extraction temperature (°C)	Extraction time (min)	Experimental (mg/g)	Predicted (mg/g)
3.9	36.25	39.40	21.3	5.76 ± 0.41	5.78 ± 0.21

Extraction optimization results showed that water can serve as an efficient solvent alone with UAE treatment as it can extract comparable yields of gallic acid. A study reported a gallic acid yield of 156 mg/100 g of MSK through shaking in 80% methanol (Abdel‐Aty et al., 2018), which is too low in comparison to our findings. However, the optimized yield of gallic acid is slightly lower than the gallic acid content quantified as 6 mg/g in MSK extracts obtained by using 95% methanol, as reported by Abdalla et al. (2007), as well as our previous work, where the optimized gallic acid yield was 6.11 ± 0.11 mg/g obtained by using 19.4% ethanol (Hayat et al., 2023). The gallic acid yield of the current study was higher when compared with conventional decoction extraction of raw MSK reported in a previous study by our research group, which yielded 2.03 ± 0.01 mg/g of gallic acid (Hayat et al., 2023). Even so, the utilization of higher amounts of organic solvents is important to consider their environmental impact as well as the cost of the extraction process. When it comes to considering the cost effectiveness of solvents, using water as a solvent can be a great alternative to organic solvents. Water is a cheap and widely available solvent that can be used in a variety of extraction processes. Furthermore, using water as a solvent is a more sustainable option, since it does not have the same environmental impact as organic solvents (Alonso‐Riaño et al., 2020). It's important to consider the specific needs of each extraction process, but overall, using water as a solvent can be a cost‐effective and sustainable choice. Therefore, this green extraction of MSK to get gallic acid‐enriched extracts may be cost‐effective in terms of cutting down the cost involved in organic solvents as well as being environment friendly. It may help in designing more efficient extraction methods for the valorization of fruit waste to extract this valuable compound, especially from mango waste, for its therapeutic utilization and food applications.

## 4 CONCLUSION

Mango seed kernel is a bioactive compound, and if utilized properly, it can serve as a potent nutraceutical. The current study was aimed at optimizing gallic acid extraction by using MSKs through UAE with water as a solvent. The variables for extraction were finalized on the basis of single‐factor extraction, and optimization was undergone via RSM. Under the optimal conditions of 3.9 pH, 36.25 mL/g solvent ratio, 39.4°C extraction temperature, and 21.3 min, the yield of gallic acid obtained was 5.76 ± 0.41 mg/g, which is comparable to the solvent‐extracted yield of gallic acid from MSK. Hence, it proves that the study is not only one of its kind, but it also provides experimental information about any organic solvent‐free extraction of a bioactive compound from agro‐waste by using UAE. The valorization of agro‐industrial waste by using UAE to obtain gallic acid, which is safe for its utilization as a potential nutraceutical along with its other beneficial properties, is possible through the upscaling of this method. However, more research is required to explore this reported process with combinations of other extraction techniques that might enhance the yield of gallic acid without using hazardous solvents.

## AUTHOR CONTRIBUTIONS

**Tuba Riaz:** Conceptualization (equal); data curation (equal); formal analysis (equal); methodology (equal); software (equal); validation (equal); visualization (equal); writing – original draft (equal); writing – review and editing (equal). **Zafar Hayat:** Conceptualization (equal); funding acquisition (equal); investigation (equal); methodology (equal); project administration (equal); supervision (equal); validation (equal); visualization (equal); writing – review and editing (equal). **Kinza Saleem:** Conceptualization (equal); data curation (equal); formal analysis (equal); methodology (equal). **Kashif Akram:** Conceptualization (equal); methodology (equal); software (equal); visualization (equal); writing – review and editing (equal). **Hafeez Ur Rehman:** Methodology (equal); software (equal); validation (equal); visualization (equal). **Shafiq ur Rehman:** Funding acquisition (equal); project administration (equal); supervision (equal). **Muhammad Azam:** Formal analysis (supporting).

## FUNDING INFORMATION

This work was carried out with financial support from the UK government Department of Health and Social Care (DHSC), the Global AMR Innovation Fund (GAMRIF), and the International Development Research Centre Ottawa, Canada (Grant No. 109051‐003). The views expressed herein do not necessarily represent those of IDRC or its Board of Governors.

## CONFLICT OF INTEREST STATEMENT

The authors declare no conflict of interest.

## Data Availability

The data that support the findings of this study are available from the corresponding author upon reasonable request.
